# Production of Biodiesel from *Chlorella* sp. Enriched with Oyster Shell Extracts

**DOI:** 10.1155/2014/105728

**Published:** 2014-02-17

**Authors:** Cheol Soon Choi, Woon Yong Choi, Do Hyung Kang, Hyeon Yong Lee

**Affiliations:** ^1^Department of Biomedical Materials Engineering, Kangwon National University, Chuncheon 200-701, Republic of Korea; ^2^Korea Institute of Ocean Science & Technology (KIOST), P.O. Box 29, Ansan, Seoul 426-744, Republic of Korea; ^3^Department of Food Science and Engineering, Seowon University, Cheongju 361-742, Republic of Korea

## Abstract

This study investigated the cultivation of the marine microalga *Chlorella* sp. without supplying an inorganic carbon source, but instead with enriching the media with extracts of oyster shells pretreated by a high-pressure homogenization process. The pretreated oyster shells were extracted by a weak acid, acetic acid, that typically has harmful effects on cell growth and also poses environmental issues. The concentration of the residual dissolved carbon dioxide in the medium was sufficient to maintain cell growth at 32 ppm and pH 6.5 by only adding 5% (v/v) of oyster shell extracts. Under this condition, the maximum cell density observed was 2.74 g dry wt./L after 27 days of cultivation. The total lipid content was also measured as 18.1 (%, w/w), and this value was lower than the 23.6 (%, w/w) observed under nitrogen deficient conditions or autotrophic conditions. The fatty acid compositions of the lipids were also measured as 10.9% of C16:1 and 16.4% of C18:1 for the major fatty acids, which indicates that the biodiesel from this culture process should be a suitable biofuel. These results suggest that oyster shells, environmental waste from the food industry, can be used as a nutrient and carbon source with seawater, and this reused material should be important for easily scaling up the process for an outdoor culture system.

## 1. Introduction

The increasing consumption of fossil fuels is leading to an energy depletion crisis, and the carbon dioxide arising from the use of fossil fuels is impacting the global environment day by day [1, 2]. In particular, the development of bioenergy using marine and freshwater microalgae as a 3rd generation biomass feedstock has been highlighted [3] because microalgae grow fast and have an excellent CO_2_ absorption capacity as well as a relatively easy to control lipid and sugar content under various culture conditions [4–7]. However, in general, the production cost of fuel from microalgae or other marine bioresources is more than two times as high as fossil fuel-derived diesel. To solve this problem, many researchers are trying to lower production costs by screening microalgae that have a high lipid content, performing genetic manipulation on microalgae, minimizing the cost of microalgae culture processes, and so on [8–10]. In other reports, there have been several attempts to develop relatively cheap organic carbon sources, such as acetate for mixed cultures of *Chlorella sorokiniana*, instead of autotrophic cultivation [11–14].

Therefore, in this work, the major portion of the culture production costs has been reduced by using significantly less artificial medium for microalgae growth [15], which is an expensive material. Additionally, we are interested in recycling oyster shells, a waste resource that has caused serious sea pollution, such as the corruption of parasitic organisms and the mass proliferation of pathogenic bacteria [16]. As shown in Table 1, oyster shells contain high amounts of essential minerals and 2.7 g of organic matter per 1 kg and can also serve as an inorganic carbon source by properly treating the calcium carbonates in the oyster shells [17]. This waste material is a potential substitute for expensive essential medium supplements, such as F/2 mixtures in seawater media for microalgal growth [18, 19]. However, CaCO_3_ dissolves in HCO^3-^, CO_3_^2−^, and H_2_CO_3_ only under acidic conditions and is ultimately converted to CO_2_ [20]. For the acidification of oyster shell media, the media need to be made under acid conditions; however, a large amount of acid cannot be added at a given time due to environmental concerns.

Therefore, developing a system to facilitate an increased reaction under mild acid conditions by decomposing the oyster shells is essential. Among the many methods to pulverize oyster shells, a high-pressure homogenization pretreatment process can be applied, which can favorably produce more CaCO_3_, HCO^3−^, CO_3_^2−^, and H_2_CO_3_ because the oyster shells are broken down to less than 5 *μ*m in size, greatly increasing the surface area of the oyster shells. Oyster shells with a small particle size can easily be decomposed, even by the treatment of a weak acid, such as acetic acid (CHCOOH), and not strong acids, such as sulfuric acid (H_2_SO_4_) and hydrochloric acid (HCl). Another advantage of using high-pressure homogenization is that small particle sizes are obtained from hard materials through a simple and short process, which is a great benefit in scaling up the pretreatment process. Then, the oyster shells, a marine waste material, can be recycled as they are an excellent resource that supplies both inorganic carbon and essential nutrients. The supply of dissolved carbon dioxide from the culture medium will be the most important consideration in overcoming the limits of scaling up the outdoor culture system because the external supply of carbon dioxide is one of the major bottlenecks for large-scale outdoor culture systems [21, 22].

## 2. Materials and Methods 

### 2.1. Materials

*Chlorella *sp. was obtained from the Korea Marine Microalgae Culture Center. *Chlorella *sp. was cultured, and then the scaled-up 14 L volume was inoculated at 10% (v/v). 10 w/m^2^ of light was applied using a 20 W fluorescent lamp as the light source. The culture conditions were maintained at a stirring speed of 180 rpm and a temperature of 28°C in a 14 L shaking incubator (KF-20 L CONTROL PANEL, KOREA). The cultured cells were filtered through a 0.4 *μ*m filter paper (Whatman, number 1, England) and centrifuged at 3000 rpm for 10 min, and the supernatant of the medium was removed; only the cells in the lower layer were freeze-dried. This sample was sealed and refrigerated at 4°C. The oyster shells supplied as a nutrient source in the culture medium were from Taejon, South Chungcheong Province, South Korea. The collected oyster shells were washed 3 times using DW (distilled water), which removed dirt and impurities. The naturally dried oyster shells were dried again for 24 hours in a dry oven at 105°C and were pulverized using a pestle and a mortar. Using a mesh of 1 mm or less in particle size, pulverized oyster shells were filtered from the sample and used in the experiment. For the basal medium for *Chlorella *sp. growth, f/2 medium was purchased from Sigma (St. Louis, USA) and dissolved in 1 L of seawater. The detailed composition of the f/2 and water mixture was as follows: 29.23 g NaCl, 1.105 g KCl, 11.09 g MgSO_4_·7H_2_O, 1.21 g Tris-base, 1.83 g CaCl_2_ 2H_2_O, 0.25 g NaHCO_3_, and a 3.0 mL trace metal solution that was composed of 281.3 mg NaNO_3_, 21.2 mg NaH_2_PO_4_·H_2_O, 16.35 mg Na_2_·EDTA, 11.8 mg FeCl_3_·6H_2_O, 675 *μ*g MnCl_2_·4H_2_O, 37.5 *μ*g CoCl_2_·6H2O, 37.5 *μ*g ZnSO_4_·7H_2_O, 22.5 *μ*g Na_2_MoO_4_, 0.375 mg vitamin B1, and 0.188 of *μ*g biotin [23]. In addition, for the nitrogen deficient medium, the amount of NaNO_3_ in the above basal medium was reduced to 37.5 mg. The remaining composition was prepared and used in the same way as the f/2 medium.

### 2.2. Pretreatment of Oyster Shells

The pretreatment process for the oyster shells medium using a high-pressure homogenizer (High-pressure Processor; MN400BF; PiCOMAX, Korea) was as follows: oyster shells in the form of dry powder were added to 1 L of distilled water at concentrations of 1, 5, and 10 (%) and were shred for 15–20 minutes at 20,000 rpm by a high-speed grinder. These shells were crushed to a size of approximately 50~70 *μ*m. For the high-pressure homogenization process, the crushed powder was applied at 20,000~25,000 psi of high pressure and recycled two times for 30 min of total operation time, where the oyster shells were broken down to less than 5 *μ*m in particle size, as observed with DLS (US/90Plus, Brookhaven Instruments Co., USA). Then, the particle powder was dissolved in 1 N acetic acid. After dissolving the powder, 1%, 5%, or 10% (v/v) of the carbonate solution was mixed with the f/2 containing basal culture medium and filtered using a vacuum filtration device to prepare the final culture medium. The pH of final medium was measured to calculate dissolved carbon dioxide concentrations in the medium by (1) and was estimated as 6.8, 6.7, and 6.5 for the cases of adding 1%, 5%, and 10% oyster shell solutions, respectively.

### 2.3. Experimental Design of Cultivation

A 5 L photobioreactor was illuminated with 10 w/m^2^ of light intensity using a 20 W fluorescent lamp at 28°C and 180 rpm of agitation speed. In the photobioreactor, 3 L of basal medium enriched with 1, 5, and 10 (%, v/v) of the dissolved oyster shell solution was added, without additional CO_2_. As the control groups, f/2 medium with and without 5% (v/v) carbon dioxide gas that was added to the photoreactor was also used for growing *Chlorella* sp. As a positive control, basal medium enriched with a 5% oyster shell solution was treated by hydrochloric acid, maintaining a pH of 6, and was used to grow *Chlorella* sp. For nitrogen deficient growth, after 15 days of cultivation with the f/2 medium enriched with the 5% oyster shell solution but without supplying carbon dioxide, the medium was drained through two drain pipes at the bottom of the reactor that had a 0.45 um membrane filters installed. Then, nitrogen deficient medium containing 37.5 mg/L NaNO_3_ in the basal medium was added to the reactor, and the cells were continuously grown for the rest of the cultivation.

### 2.4. Measurement of the Cell Density and Dissolved CO_2_ Concentrations

To measure the cell growth of *Chlorella *sp. in the medium, cells were taken every 3 days from the reactor under the different experimental conditions. Then, the absorbance was measured at a wavelength of 682 nm using a UV/Visible spectrophotometer (Kontron Instruments, Germany), and cell mass was estimated by using calibrated standard curves [24]. For the measurement of dry cell mass, centrifugation was carried out for 15 minutes at 12,000 rpm. The cells were washed 2 times using distilled water and dried at 105°C for 24 hours, and then the dry matter was weighed to determine the dry cell density [25, 26]. The dissolved CO_2_ concentration, a carbon source in the medium, was calculated by (1) because, during acid-base equilibrium, pH controls the relative concentrations of each species in the inorganic carbon system [27]:

(1) $$\begin{array}{c} \left[\text{pC}{\text{O}}_{2}\right]=\frac{\left[\text{DIC}\right]}{\left(1+K_{0}K_{1}/\left[{\text{H}}^{+}\right]+K_{0}K_{1}K_{2}/{\left[{\text{H}}^{+}\right]}^{2}\right)}, \end{array}$$

where the equilibrium constants were *K*_0_ = [H_2_CO_3_]/[CO_2_] (≅1.58 × 10^−3^), *K*_1_ = [H^+^][HCO_3_^−^] (≅2.83 × 10^−4^ amol L^−1^), and *K*_2_ =/[H_2_CO_3_] [CO_3_^2−^][H^+^] (≅4.68 × 10^−11^ amol L^−1^); square brackets indicate molar concentration.

### 2.5. Measurement of Total Lipid Contents and Fatty Acid Profiles

For the measurement of the lipid content in cells, the Folch method was used. In a 1 g sample of dry cells, a 20 mL solution of CHCl_3_ : methanol (2 : 1, v/v) was added, and the solution was mixed. The mixture was stirred for 30–90 minutes at room temperature and then centrifuged for 15 minutes at 500 rpm. Then, the supernatant was removed, and a 0.9% (w/v) NaCl solution was added to the pelleted cells. After vortexing the mixture for a few seconds, centrifugation was carried out for 15 minutes at 2000 rpm. The supernatant was discarded and the lower fluid layer that contained lipids was dried. The dried fluid was weighed for the determination of the fat content [28, 29].

After the culture was terminated, on the 27th day the fatty acids from the lipids were analyzed by the Folch method. The methyl esterification of the lipids was evaluated by gas chromatography (HP 6890 SERIES, USA) analysis. Methylation was conducted by applying 0.5 N NaOH/MeOH to the obtained lipids. Then, 1.5 mL of BF3-methanol was added, and the solution was reacted at 95°C for 60 minutes. After methyl ester transesterification, 1 mL of hexane was added, and the solution was mixed well. The supernatant (hexane layer) was removed and then analyzed by GC. As a GC detector, a flame ionization detector (FID) was used. Additionally, a SP-2560 GC column (100 m × 0.25 mm × 0.2 *μ*m, number 24056, Supelco, USA) was used. The oven temperature was changed at a rate of 4°C /min from 240°C to 100°C. The injection temperature and detector temperature were set to 250°C and 280°C, respectively. The flow rate was set to 1.0 mL/min and the split ratio to 50 : 1. Lastly, fatty acid analysis was performed by injecting 1 *μ*L for each sample [30].

## 3. Results and Discussion

### 3.1. Comparison of Cell Growth in Different Media

**Figure 1:**
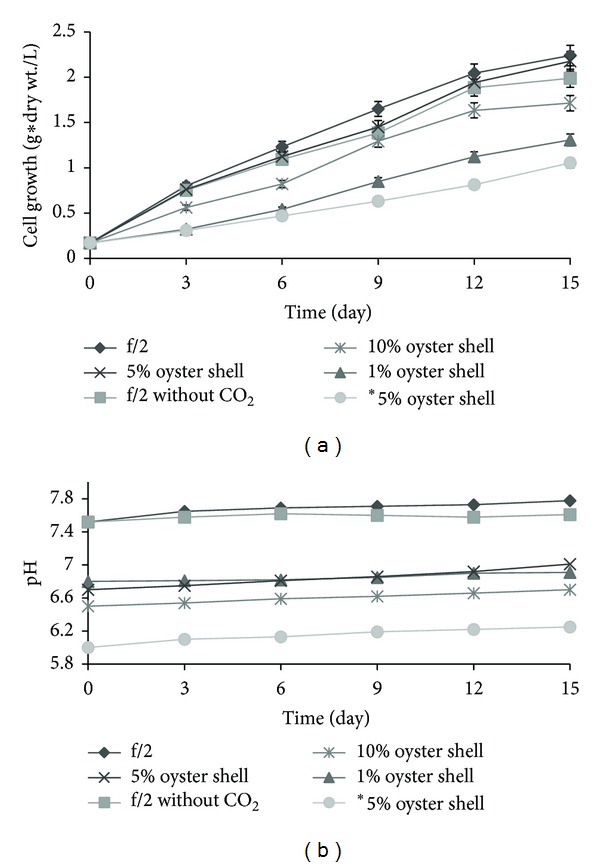
Comparison of cell growth and pH under different culture medium conditions. 1% oyster shells pretreated with a high-pressure homogenization process and acetic acid (pH 6.8); 5% oyster shells pretreated with a high-pressure homogenization process and acetic acid (pH 6.7); 10% oyster shells pretreated with a high-pressure homogenization process and acetic acid (pH 6.5); *5% oyster shells pretreated with a high-pressure homogenization process and hydrochloric acid (pH 6).

Figure 1 shows the comparative results of cell growth and pH for several different culture media. A higher cell density of 2.24 g dry wt/L was obtained for cells growing in f/2 with additional carbon dioxide compared to 2.17 g/L for cells with medium containing 5% oyster shell extracts treated with acetate because of the mild pH of 6.7. On the other hand, for the case of adding 10% or 1% of oyster shells to the media, relatively low cell growth was maintained due to the low pH required for optimal cell growth and the low amounts of essential elements in the medium, respectively, which resulted in a small pH increase during the cultivation. Low amounts of key elements, such as nitrogen and phosphorus, are present in seawater and thus increase the growth of microalgae in combination with the oyster shells [18]. Low concentrations of carbon supply were observed to have little effect on the growth of microalgae. This result was also confirmed by reports that cell mass and productivity decreased because the carbon sources acted as a stressor to the microalgae under conditions where the carbon sources were excessively supplied [31, 32].

In this study, the pH of the medium was varied by adding acetic acid or hydrochloric acid to increase the amount of CaCO_3_-derived carbon sources (initial pCO_2_), an important component in increasing the growth of *Chlorella *sp. The composition of the oyster shell medium affects how much CaCO_3_ reacts with CO_2_ and H_2_O and subsequently exists as dissolved ions, such as HCO^3−^, CO_3_^2−^, and H_2_CO_3_. Then, it was converted to CO_2_. Therefore, this acid molecule lowers the pH and has an impact on the lipid metabolism and growth of microalgae [23].  Table 2 shows the relationship between the initial pH and dissolved carbon dioxide (pCO_2_) that supplies the main carbon source for cell growth. The initial amount of pCO_2_ was expected to gradually increase, lowering the pH, which would cause cell growth to increase. However, at a low pH that can maintain a high concentration of dissolved carbon dioxide, cell growth did not increase much, as shown in Figure 1, because the environmental stress due to the lowered pH caused negative consequences for the growth of *Chlorella *sp. [33]. Additionally, the 5 *μ*m sized oyster shells produced by nanogranularization and high-pressure homogenization before treatment and the change in pH were confirmed to increase the initial carbon dioxide content.

**Figure 2:**
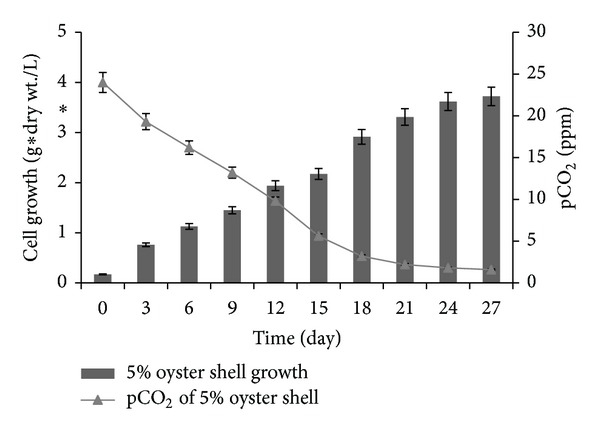
The cell growth and residual dissolved CO_2_ concentration in the medium enriched with 5% oyster shells pretreated with a high-pressure homogenization process and acetic acid (pH: 6.7).

As shown in Figure 2, the change in pCO_2_ was compared to the growth of *Chlorella *sp. in the 5% oyster shell containing medium. Little change was observed in the carbon dioxide concentration of the medium by the 12th day. However, after 15 days of cultivation, the growth of *Chlorella *sp. rapidly increased because the dissolved carbon dioxide rapidly decreased until the 24th day; thereafter, not much cell growth occurred. These results imply that large amounts of CaCO_3_ were converted to CO_2_, and this molecule was the main carbon source after decreasing the pH during the slow cell growth in the lag phase, which greatly promoted the growth of *Chlorella* sp. Therefore, the growth of *Chlorella* sp. proceeded efficiently by supplying CaCO_3_ from oyster shells without an external supply of CO_2_.

### 3.2. Cell Growth and Total Lipid Production under Nitrogen Deficiency

**Figure 3:**
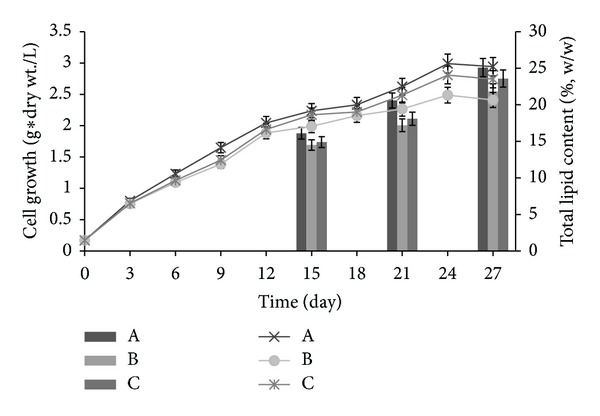
The cell growth (lines) and total lipid production (bars) obtained using different culture media. The arrow was the point where the nitrogen deficient medium was provided after the 15th cultivation day. (A: f/2 medium; B: f/2 medium without CO_2_; C: 5% oyster shells pretreated with high-pressure homogenization and acetic acid (pH: 6.7).)

In Figure 3, to increase the total amount lipid production and cell growth, *Chlorella* sp. cultured after 15 days was exposed to an insufficient nitrogen source (from 400 mg to 37.5 mg of NaNO_3_), based on previous experiments [34, 35]. Cell growth was compared between one case where the pH of the 5% oyster shell medium was titrated to 6.7, the most efficient pH for culturing *Chlorella* sp., and another case with the f/2 medium as the control group, where more CO_2_ was not added. When cells were grown with f/2 medium and shifted to the nitrogen deficient medium, higher cell density was observed and 2.94 g dry wt/L compared to 2.74 g/L of cell density was observed from the 5% oyster shell enriched medium (pH 6.7). In addition, the maximum cell density, 2.41 g/L, was observed for the f/2 medium without an additional CO_2_ supply. The results confirmed that when cells were shifted to the nitrogen deficient medium with the 5% oyster shell enriched medium, relatively good cell growth could be maintained without supplying a carbon source. However, cell growth when cells were initially exposed to nitrogen deficient medium did not increase significantly compared to the cell growth increase observed in previous experiments. These results suggest that insufficient nitrogen sources slow down the growth of *Chlorella* sp. A similar investigation also showed that an adequate supply of nitrogen sources was essential to promote the growth of the microalgae *Chlorella* sp. [34].

In addition to cell growth, when shifting from the normal medium to the nitrogen deficient medium, the lipid contents were greatly increased, comparing the 15th day and the 27th (last) day of culture. For the artificial f/2 medium, the lipid content was 20.6 (%, w/w) on the 21st day and 25.1 (%, w/w) on the 27th day. The 5% oyster shell enriched medium (pH 6.7) was 18.1 (%, w/w) on the 21st day and 23.6 (%, w/w) on the 27th day. Interestingly, in the seawater with f/2 medium where additional CO_2_ had not been added, the lipid content was estimated as only 17.2 (%, w/w) on the 21st day and 21.8 (%, w/w) on the 27th day. In general, the total lipid contents exhibited a large increase of 3~5 (%, w/w) after shifting to the nitrogen deficient medium and an especially large increase when grown in the oyster shell enriched medium. This result also implied that low pH acted as an environmental stress on *Chlorella* sp.; therefore, brisk cell lipid metabolism occurred and led to an increase in lipid accumulation [36, 37]. These results indicate that, in culture under stressful conditions, the metabolic changes resulted in an increase in lipid content, rather than the growth of *Chlorella* sp. [34]. Additionally, cells growing in the 5% oyster shell enriched medium exhibited a lipid content under the nitrogen deficient condition that was not much different from the lipid content of cells grown in the artificial seawater f/2 medium. This result also confirmed that oyster shells can be used for the growth of *Chlorella* sp. as well as increasing the total lipid content more efficiently without an additional supply of carbon, although a higher lipid content was observed in the control group.

### 3.3. Production of Total Lipids and the Fatty Acid Profiles of Algae from the Oyster Shell Enriched Medium

Based on the results in Figure 3, lipid productivity from each medium was estimated in Table 3, and the identities of the fatty acids in the total lipid obtained from each medium are also displayed. For 21 days of cultivation (from the artificial seawater f/2 medium to the nitrogen deficient medium), the lipid productivity was 25.5 mg/L/day, which was lower than the 28.8 mg/L/day productivity from 27 days of cultivation where longer nitrogen depletion was maintained. For the case of adding a 5% oyster shell enriched medium, the lipid productivity was 20.6 mg/L/day after 21 days of cultivation and 25.3 mg/L/day after 27 days of cultivation. Interestingly, in the f/2 medium control group without a CO_2_ supply, the lowest lipid productivities of 19.2 mg/L/day and 23.4 mg/L/day were observed for 21 days and 27 days of cultivation, respectively. A minimal difference in the lipid productivity between the f/2 medium with CO_2_ supply and 5% oyster shell enriched medium without CO_2_ supply was also confirmed. Thus, the possibility of promoting the continued growth of cells and an adequate lipid content in the absence of an expensive artificial seawater f/2 medium was demonstrated if oyster shells and appropriate nitrogen sources were supplied. Nitrogen induced an increase in the lipid content of the cell and a maximum amount of cell. Several factors contributed to these effects: cells were switched to a nitrogen deficient medium, the pH was increased after the acid treatment of the oyster shells, and the oyster shells underwent a high-pressure homogenization before treatment.

To compare the quality of biodiesel from each lipid, the fatty acid composition of the total lipids obtained from each medium is also shown in Table 3. The fatty acid content of the total lipids from the f/2 medium showed that the C14:0, C16:0, and C18:0 saturated fatty acids were 12.5, 13.8, and 11.8 (%, w/w), respectively, which are the highest observed values. For the case from the 5% oyster shell enriched medium, the results showed 11.5, 12.5, and 11.2 (%, w/w), respectively. In the case of the f/2 medium without a CO_2_ supply as the control group, the results showed 10.8, 11.8, and 10.7 (%, w/w), respectively.

For monounsaturated fatty acids, C16:1, the lipid content in the f/2 medium, 5% oyster shell medium, and f/2 medium without CO_2_ supply was 12.1, 10.9, and 11.5 (%. w/w), respectively. However, the C18:1 lipid content in the f/2 medium, 5% oyster shell medium, and the f/2 medium without CO_2_ supply was 17.5, 16.4, and 17.1 (%. w/w), respectively. In the case of polyunsaturated fatty acids, the C18:2 and C18:3 lipid content in the f/2 medium were 20.6 and 13.4 (%, w/w), respectively. The C18:2 and C18:3 fatty acids were also present in the 5% oyster shell enriched medium, reaching values of 18.5 and 12.3 (%, w/w), respectively. Lastly, the C18:2 and C18:3 fatty acids were 19.5 and 10.8 (%, w/w), respectively, of the fatty acid content for the f/2 medium without CO_2_ supply. In general, monounsaturated fatty acids, such as C16 and C18, are known to be suitable as fatty acids for biodiesel [38], and in this work, the total content of C16:1 and C18:1 from the lipids of *Chlorella *sp. was estimated as ca. 30 (%, w/w), showing almost the same results for both the f/2 medium and the 5% oyster shell enriched medium. Therefore, the oyster shell culture medium has several advantages, including representing a cheaper carbon source than the commonly used carbon sources, such as sodium bicarbonate, among others, and does not require an additional supply of CO_2_, which will have economic benefits for the scaleup needed for biodiesel production.

## 4. Conclusion 

To overcome economic limitations for producing biodiesel from microalgae, this study cultivated the marine microalga *Chlorella* sp. with simple seawater enriched with 5 (%, v/v) of high-pressure homogenized oyster shells. This medium was observed to continuously provide an inorganic carbon source without adding external inorganic carbon sources, which has represented a bottleneck in scaling up the culture process, especially for outdoor mass cultivation. The high-pressure homogenization helped weak acids to easily dissolve the hard oyster shells, eliminating the need for strong acids due to the increase in the surface areas and mechanical stresses on the surfaces of the hard shells. When this optimal basal medium was changed to a nitrogen deficient medium during cultivation, 2.74 g/L of dry cell density and 23.6 (%, w/w) of total lipid content were obtained. These results were better than a previous investigation using Tris-acetate-phosphorus (TAP) medium, which had 1.76 g/L of dry cell density [39]. Nitrogen deficient conditions were confirmed to dramatically increase the total lipid contents observed, from 18.1 (%, w/w) to 23.6 (%, w/w), but to significantly decrease cell growth. This result implied that better lipid productivity obtained using this medium could be maintained for longer-term cultivation than was possible for the conventional enriched f/2 medium. Fatty acid profiles of the lipids obtained from this oyster containing medium were not much different from the f/2 enriched medium, showing that ca. 25% of the total fatty acids were C16:1 and C18:1 (key fatty acids for bio-diesel production) for cells from the f/2 culture medium, while ca. 23% of the fatty acids were these key fatty acids for cells from the 5% oyster shell containing medium. These results confirmed that this unique inexpensive medium has great advantages for reducing environmental issues and for enhancing both cell growth and lipid production under proper culture conditions.

## Figures and Tables

**Table 1:** Comparison of the chemical compositions of f/2 medium and 5% oyster shells with seawater.

Chemical composition		Oyster shell (g/L)	f/2 (g/L)
Al_2_O_3_		0.0042	—
CaCO_3_		0.959	—
MgO		0.0065	—
NaCl		—	29.23
KCl		—	1.105
MgCl		1.83	—
Na_2_O		0.0098	—
MgSO_4_·7H_2_O		0.048	11.09
NaHCO_3_		0.25	0.25
Tris-base		—	1.21
NaNO_3_		—	0.281
NaH_2_PO_4_·H_2_O		—	2.12 × 10^−2^
Na_2_EDTA		—	1.635 × 10^−2^
FeCl_4_·6H_2_O		—	1.18 × 10^−2^
MnCl_2_·4H_2_O		—	6.75 × 10^−8^
CoCl_2_·6H_2_O		—	3.75 × 10^−5^
ZnSO_4_·7H_2_O		—	3.75 × 10^−5^
Na_2_MoO_4_		—	2.25 × 10^−5^
Vitamin B1		—	3.75 × 10^−4^
Biotin		—	1.88 × 10^−7^
CaCl_2_·2H_2_O		—	1.83

Seawater (mol/kg)	Cl^−^	0.546	
	Na^+^	0.469	
	Mg^2+^	0.528 × 10^−1^	
	SO_4_^2−^	0.282 × 10^−1^	
	Ca^2+^	0.103 × 10^−1^	
	K^+^	0.102 × 10^−1^	
	Br^−^	0.844 × 10^−3^	
	Sr^2+^	0.91 × 10^−4^	
	F^−^	0.68 × 10^−4^	
	N_3_^−^	0.11 × 10^−2^	
	P^3−^	2.84 × 10^−6^	

**Table 2:** Comparison of the initial dissolved CO_2 _concentrations in several culture media according to the initial pH.

Parameter	Culture medium			
	A	B	C	D
pH value	6.8	6.7	6.5	6
Dissolved CO_2_ (ppm)	22	24	32	108

A: 1% oyster shells pretreated with a high-pressure homogenization process and acetic acid; B: 5% oyster shells pretreated with a high-pressure homogenization process and acetic acid; C: 10% oyster shells pretreated with a high-pressure homogenization process and acetic acid; D: 5% oyster shells pretreated with a high-pressure homogenization process and hydrochloric acid.

**Table 3:** Results of estimating lipid productivity and the fatty acid profiles of cells grown from several culture media under different culture conditions.

			Culture time					
Parameter			21 days (6 days of N depletion)			27 days (12 days of N depletion)		
			A	B	C	A	B	C
	Lipid productivity (mg/L/day)		25.5	19.2	20.6	28.8	23.4	25.3

Culture medium	Total lipids (%, w/w)	Fatty acids composition (% of total fatty acids)						
		C_14:0 _	C_16:0_	C_16:1_	C_18:0_	C_18:1_	C_18:2_	C_18:3_

A	25.1	12.5	13.8	12.1	11.8	17.5	20.6	13.4
B	21.8	10.8	11.8	11.5	10.7	17.1	19.5	10.8
C	23.6	11.5	12.5	10.9	11.2	16.4	18.5	12.3

A: F/2 medium; B: F/2 medium without CO_2_; C: 5% oyster shells pretreated with high-pressure homogenization and acetic acid (pH: 6.7).
